# Rubella virus-associated granulomas controlled with allogeneic hematopoietic stem cell transplantation

**DOI:** 10.1007/s10875-024-01756-z

**Published:** 2024-07-04

**Authors:** Timo Hautala, Ludmila Perelygina, Urpu Salmenniemi, Mikko R. J. Seppänen, Rubella consortium, Eeva Martelin, Vesa Lindström, Jouni Heiskanen, Terttu Harju, Airi Jartti, Päivi Jackson, Kaisa Tasanen, Outi Kuismin, Hannu Tuominen, Kathleen E. Sullivan, Yenan Bryceson

**Affiliations:** 1 Research Unit of Internal Medicine and Biomedicine, University of Oulu, FIN-90014 Oulu, Finland; 2 ERN-RITA Core Center Member, RITAFIN Consortium, Infectious Diseases Clinic, Oulu University Hospital, Oulu, Finland; 3 Centers for Disease Control and Prevention, Division of Viral Diseases, Atlanta, GA USA; 4 Department of Hematology, Comprehensive Cancer Center, Helsinki University Hospital and University of Helsinki, Helsinki, Finland; 5 ERN-RITA Core Center Member, RITAFIN Consortium, Rare Disease Center and Pediatric Research Center, New Children’s Hospital, University of Helsinki and HUS Helsinki University Hospital, Helsinki, Finland

**Keywords:** Rubella virus, Immunologic Deficiency Syndromes, Hematopoietic Stem Cell Transplantation, Inborn Errors in Immunity, Granulomatous inflammation

To the Editor,

The live attenuated vaccine to prevent infection with rubella virus (RuV) has been associated with chronic granulomas in patients suffering from inborn errors in immunity (IEI). Cutaneous RuV positive granulomas have been reported in syndromic DNA repair disorders and occasionally in other forms of IEI syndromes. [1–3] Replication and evolution of the vaccine-derived RuV in IEI patients resulted in mutated viruses and persisting infection primarily observed in macrophages. [4] While a small number of pediatric patients has undergone allogeneic hematopoietic stem cell transplantation (HSCT) and received selected antiviral medications with limited success, there is still a need to demonstrate uniformly effective treatment for the RuV granulomas. [5]

A previously healthy female patient with non-consanguineous parents presented with facial skin lesions at the age of 15 years (Fig. 1 A1). The history of the patient or her family members was negative for recurrent, severe, or opportunistic infections, notable travel history, exposure to harmful infectious or chemical substances, or immune-mediated diseases. Her growth and development had been uneventful. She had received routine measles, mumps, and rubella (MMR) vaccinations at the ages of 18 months and six years. From age 16 to 17 years, the initially inconspicuous cutaneous facial lesions progressed aggressively into granulomas (Fig. 1 A2-3). Despite extensive diagnostic testing, all microbiological samples including mycobacteria consistently yielded negative results. Responses to both local and systemic antimicrobials were, at best, modest.

**Fig. 1:**
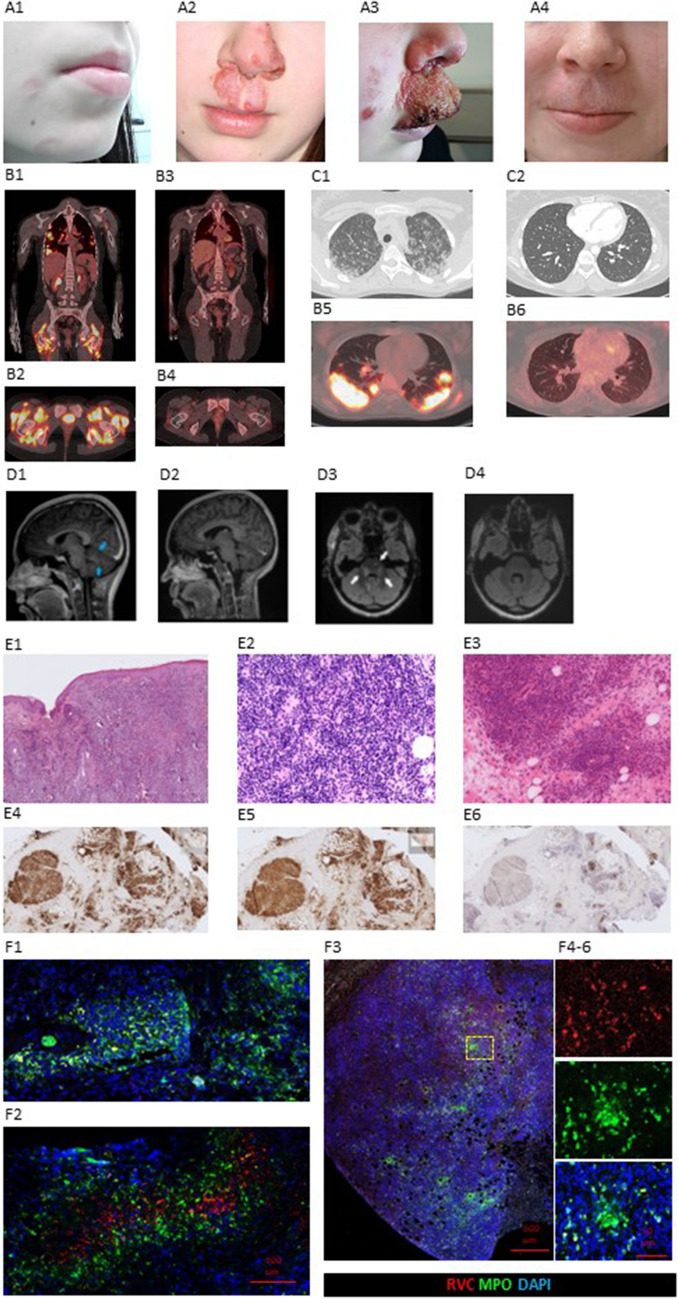
Clinical progression of skin granulomas before (**A1-3**) and one year after (**A4**) hematopoietic stem cell transplantation (HSCT). Positron emission tomography-computed tomography (CT) before (**B1-2**, **B**5) and one year after (**B3-4**, **B6**) HSCT. Chest CT before (**C1**) and one year after (**C2**) HSCT. Brain magnetic resonance imaging before (D1, D3), and one year after (**D2**, **D4**) HSCT. Skin granulomatous histology (**E1**) with lymphocytes infiltrates (**E2**). Quadriceps femoris muscle biopsy with severe atrophy and granulomatous infiltrates (**E3**) presenting with CD3^+^ (**E4**) CD4^+^ (**E5**) and CD20 negative (**E6**) lymphocytes. Rubella Virus capsid (RVC) positive findings in skin (**F1**, **F2**) and muscle (**F4-6**) mainly in MPO+ neutrophils

Gradually, she developed additional symptoms, including lymphadenopathy, lower limb weakness, central nervous system (CNS) symptoms (headache, involuntary movements, disturbed vision), and respiratory distress. Chest computed tomography (CT) showed extensive pulmonary infiltration and enlarged lymph nodes (Fig. 1 C1). Later, a cavitating lesion in the right lung was surgically removed. Magnetic resonance imaging (MRI) revealed lesions in the brain (Fig. 1 D1,D3) and the spinal cord. Positron emission tomography-computed tomography (PET-CT) revealed extensive activity in the CNS, lymph nodes, muscles, and lungs (Fig. 1 B1,B2,B5). Tissue samples were taken from the facial skin, axillary lymph nodes, quadriceps femoris muscle, and the lesions of the right lung. Skin histology showed extensive granulomas with lymphocytic infiltrates (Fig. 1 E1,E2). In lymph nodes, multiple CD20^+^, CD23^+^, CD3^+^ granulomas with normal levels of CD30, Bcl-6, Mum-1 were observed; immunostaining for Epstein-Barr virus and cytomegalovirus was negative. Muscles showed severe atrophy with epithelioid cell granulomas and lymphocytic CD3^+^CD4^+^ infiltrates (Fig. 1 E3-E6). Lung histology was consistent with purulent infection and CD3^+^ and CD5^+^ lymphocytic infiltrates. Tissue samples were analyzed for RuV capsid protein (RVC) by fluorescent immunohistochemistry. [4] In skin, numerous RVC+/MPO+ neutrophils possibly originating from RuV persistence in myeloid precursor cells were found. Sporadic RVC+/CD68+ macrophages were detected in skin (Fig. 1 F1,F2) and muscle (Fig. 1 F3-6). However, lymph node and lung samples tested negative for RVC. Serum RuV IgG was positive (>10IU/mL) and RuV IgM was negative. The persisting RuV is highly likely a vaccine-derived strain, as 2 doses of MMR vaccine induce very efficient, life-long immunity that prevents infection with wild-type RuV strains.

Peripheral blood lymphocyte (0.6×10^9^/L, normal range 1.2-3.5×10^9^/L) and natural killer cell (49×10^6^/L, normal range 84-724×10^6^/L) counts were low. While the CD19^+^ B cell count was normal, the percentage of naïve B-cells (CD27^-^IgD^+^IgM^+^) (91.5%, normal range 43.2-82.4%) was increased, and those of switched memory (CD27^+^IgD^-^IgM^-^; 1.6%, normal range 6.5-29.2%) and marginal zone (CD27^+^IgD^+^IgM^+^; 3.7%, normal range 7.2-30.8%) B cells were low. Serum immunoglobulin concentrations were within normal range and responses to pneumococcal polysaccharide vaccine (Pneumovax®) were appropriate. The total CD3^+^ (488×10^6^/L; normal range 742-2750×10^6^/L) and CD3^+^CD8^+^ (59×10^6^/L, normal range 220-1129×10^6^/L) T cell counts were low. An increased proportion of naive CD3^+^CD4^+^ (77.3%) and CD3^+^CD8^+^ (85.5%) T cells was found. The CD4^+^ (11.2%) and CD8^+^ (5.4%) effector T cells were low and proportion of T helper 17 was normal. Percentage of T cell receptor (TCRab)^+^ CD4^-^CD8^-^ cells was not elevated (0.2-0.4%, normal <2.5%). NK cell degranulation following K562 cell, anti-CD16 or anti-CD3 stimulation, as well as IFN-gamma production after anti-CD3 stimulation were within normal range. Intracellular perforin and granzyme B expression also appeared normal. Tests for human immunodeficiency virus and autoantibodies were negative, C reactive peptide concentration (<10mg/L) and blood sedimentation rate were low (<15mm/hour) while plasma interleukin-2 receptor (CD25) was increased (4573 units/L, normal range 160-620). A gene panel (Primary Immunodeficiency Panel Plus, https://blueprintgenetics.com/) followed by whole genome sequencing (CentoGenome, www.centogene.com) failed to identify a definite cause. A missense *de novo* heterozygous *CASP10* c.758C>T, p.(Ser253Phe) (allele frequency 0.000002543, CADD 20.1) gene variant of unknown significance was reported. However, the genomic region surrounding this variant displays no genomic constraint (Z Score -0.49) and alpha missense in silico prediction (0.171) of the variant is benign.

Due to aggressive progression of RuV in this patient, the search for an unrelated donor for HSCT began for her at age 19. She received oral nitazoxanide and intravenous immunoglobulin treatment although their efficacy against RuV is questionable. At age 20, she received a peripheral blood stem cell graft from a well-matched (11/12, permissive mismatch at DPB1-locus) unrelated donor after a reduced toxicity conditioning regimen (fludarabine 150mg/kg and treosulfan 42 g/m^2^ total dose). Cyclosporine, short course of methotrexate (15 mg/m^2^ day +1 and 10 mg/m^2^ day +3 and +6) and anti-T-lymphocyte immunoglobulin (ATG Grafalon, 30 mg/kg as a total dose) were used as graft-versus-host disease (GVHD) prophylaxis. The immediate post-transplant period was uneventful, and her RuV-associated symptoms started to abate rapidly before immune reconstitution. Bone marrow recovery was normal and platelet and neutrophil engraftment were noted on days +12 and +17, respectively. On day +20, she was diagnosed with limited skin GVHD. She responded to methylprednisolone 1 mg/kg treatment and concurrent letermovir prophylaxis. She was discharged from hospital in good condition on day +28. Two months later, she experienced a mildly symptomatic SARS-CoV-2 infection, treated with remdesivir. On day +100, she displayed full donor chimerism in whole blood. Immunosuppression was successfully withdrawn and stopped 9 months after the transplant. She had regained her muscle strength and reported no respiratory or neurological symptoms. At present, there are no indications of transplant complications or chronic RuV infection on her skin (Fig. 1 A4). Brain MRI (Fig. 1 D2, D4), chest CT (Fig. 1 C2) and PET-CT (Fig. 1 B3-4, B6) displayed complete resolution of inflammatory activity.

RuV is associated with cutaneous granulomas but may manifest in other organs. In this case, HSCT led to complete recovery of health and immunity despite widely disseminated and long-lasting persistent infection with vaccine strain RuV. While RuV emergence in most patients is associated with syndromic monogenic IEI, our patient had not suffered from developmental abnormalities or previous hallmark signs of immunodeficiency. This suggests that immunological susceptibility to RuV persistence can be highly selective potentially resulting in widespread disease. There is an ongoing debate on whether the persistence of RuV antigen causes the formation of granulomas or exacerbates the severity of existing granulomatous inflammation. Consequently, RuV infection should be suspected in cases of any cryptic granuloma progression that deviates from the typical patterns for sarcoidosis or granulomatous infections, even in individuals with no previous health issues.

## Data Availability

Data obtained during the study are available from the corresponding author on reasonable request.
